# Learning models for classifying Raman spectra of genomic DNA from tumor subtypes

**DOI:** 10.1038/s41598-023-37303-w

**Published:** 2023-07-14

**Authors:** Giacomo Lancia, Claudio Durastanti, Cristian Spitoni, Ilaria De Benedictis, Antonio Sciortino, Emilio N. M. Cirillo, Mario Ledda, Antonella Lisi, Annalisa Convertino, Valentina Mussi

**Affiliations:** 1 Mathematical Institute, Utrecht University, Budapestlaan 6, 3584 CD Utrecht, The Netherlands; 2 Department of Basic and Applied Sciences for Engineering, Sapienza University of Rome, via A. Scarpa 16, 00161 Rome, Italy; 3 Institute for Microelectronics and Microsystems, CNR, via del Fosso del Cavaliere, 100 Rome, Italy; 4 Institute of Translational Pharmacology, CNR, via del Fosso del Cavaliere, 100 Rome, Italy

**Keywords:** Biophysics, Mathematics and computing

## Abstract

An early and accurate detection of different subtypes of tumors is crucial for an effective guidance to personalized therapy and in predicting the ability of tumor to metastasize. Here we exploit the Surface Enhanced Raman Scattering (SERS) platform, based on disordered silver coated silicon nanowires (Ag/SiNWs), to efficiently discriminate genomic DNA of different subtypes of melanoma and colon tumors. The diagnostic information is obtained by performing label free Raman maps of the dried drops of DNA solutions onto the Ag/NWs mat and leveraging the classification ability of learning models to reveal the specific and distinct physico-chemical interaction of tumor DNA molecules with the Ag/NW, here supposed to be partly caused by a different DNA methylation degree.

## Introduction

Tumor lineages are divided in multiple subtypes characterized by different cell proliferation, migration, and invasion capabilities which, on turn, determine cancer aggressiveness and metastatic potential^1,2^. The advent of molecular genetic technology based on DNA sequencing methods^3,4^ makes it possible to detect DNA mutations associated with cancer and related subtypes, but the expensive and complex enzyme based target or signal amplification procedures still prevent genetic analysis to be introduced in the routine diagnostic practice^5^. Thus, there is an urgent need of new strategies and tools for an early and accurate detection of the cancer subtypes, which will provide an essential guidance for personalized therapeutic treatments.

Recent studies have shown that the analysis of physical and mechanical properties, such as structural conformation, shape, length, can play an important diagnostic role, especially in DNA analysis, to quickly recognize changes and alterations associated with diseases and degenerative processes^6^. In fact, the physical and mechanical characteristics, closely related to the chemical structure of DNA, influence the interaction with the microenvironment and, ultimately, many cellular processes, such as replication, transcription and repair.

In addition, alterations of DNA methylation have been recognized as an important component of cancer development, through hypermethylation of tumor suppressor and DNA repair genes, and/or hypomethylation of oncogenes. DNA methylation is indeed a biological process by which CH_3_ methyl groups are covalently added to the DNA molecule, mainly at cytosine sites within CpG dinucleotides of specific gene promoters. In some cancers, such as colon cancer, the detection of hypermethylation may serve as a valuable biomarker for the disease condition^7^, so that DNA methylome mapping techniques can be exploited for clinical diagnostics and personalized treatment decisions^8–10^. Therefore, the development of innovative methods based on the analysis and recognition of DNA structural changes can represent an effective alternative or complementary approach to the complex genetic methods.

As we have already demonstrated, disordered nanostructures consisting of metal (Ag or Au) coated silicon nanowires (SiNWs) are a powerful Surface Enhanced Raman Scattering (SERS) platform, capable both to enhance the spectral signal related to specific vibrational modes of the cellular or molecular target, and to provide additional “probe-signals” associated to its physical and chemical interaction with the NWs^11–14^. This unique behavior could allow to capture diagnostic information carried by structural conformation and physical characteristics of healthy and malignant DNA molecules, upon Raman mapping of the dehydrated aqueous DNA droplets directly deposited onto the nanostructured platform, without any knowledge of the DNA sequence^15^. Here, we combine the sensing capacity of the SERS platform of disordered Ag/SiNWs with the classification ability of statistical models to make a step further and differentiate DNA from tumor subtypes, by exploiting their different DNA methylation degree.

We analyse two different phenotypes of two human cancer cell lines generally used to study melanoma and colon cancer progression. In particular, SK-MEL-28 and A375 cells have been selected as representative models for less and more aggressive phenotype of melanoma, respectively^16,17^, while CaCo-2 and HT29 as less and more methylated colorectal cancer cell models, respectively^18^ (for more details, see also^19–21^). As a control, human keratinocytes cell line (HaCaT), a reliable in vitro model for human normal skin, has been considered^22^. The analysis of Raman data coming from the drop mapping of the samples deposited on the Ag/SiNWs has been performed by using five different statistical methods.

Two of them are essentially geometric and involve the full data, that is to say, they are based on the evaluation of global characteristics of the spectra: the average and the $\ell ^2$ distance from the average. The remaining three approaches are more sophisticated, they take into consideration local properties of the spectra, also via an initial reduction of the amount of wavenumbers, and are based on the PCA analysis, the average pooling of the spectra, and the propagation of the spectra through a 1-D Convolutional Neural Network (CNN). We will address to the first two methods as the *global* ones, whereas the last three will be called *local* ones. We will show that all the strategies achieve a very high classification accuracy, close to 90%. Moreover the local methods allow, also, to identify the relevant spectral ranges that appear to be decisive for their correct classification.

In fact, the data distinction process exploits the information mainly inferred from two significant spectral regions: a low wavenumber (LW region) one, between 125 and 550 cm^−1^, containing features related to the different physico–chemical interaction of the DNA molecules with the nanowires; a second one at higher wavenumbers (HW region), between 2300 and 3400 cm^−1^, where vibrational peaks associated with the CH/CH_3_ bonds of the methyl groups are located. Our data therefore prove the remarkable capacity of the proposed bioanalytical platform to discriminate cancer subtypes, which appears to be due to the possibility it gives to probe the mechanical and conformational properties of DNA, thus also evidencing epigenetic effects, such us the variation of the methylation degree in malignant samples. Given the reversibility of the methylation process, the development of novel strategies to evicence such mechanism influencing gene expression without modification of the DNA sequence, can be extremely interesting, not only for diagnostic purposes, but also for therapy approaches.

## Materials and methods

In this section we describe the sample preparation procedures, the Raman measurements, and the statistical approaches developed to analyse the experimental data (see also^15,23^).

### Experimental procedures

#### Ag/SiNW substrate fabrication

Au catalyzed SiNWs have been grown on Si wafers by plasma enhanced chemical vapor deposition (PECVD) using SiH$_4$ and H$_2$ as precursors at a total pressure of 1 Torr and flow ratio SiH$_4$/(H$_2+$SiH$_4$), fixed to 1:10. The substrate temperature during the growth was kept at 350 °C, and a 13.6 MHz radiofrequency was used to ignite the plasma with power fixed at 5 W. A metal coating with a nominal thickness of 90 nm was obtained by evaporating an Ag film onto the SiNWs array.

#### Cell culture and DNA extraction

As skin cancer model^24^ we used the human melanoma cell lines SK-MEL-28 and A375 established from patient-derived cancer samples and routinely used in skin cancer research, which were compared to the human immortalized keratinocyte HaCaT as control health skin model. As colon cancer model, we used the cell lines CaCo-2 and HT29, which were compared to the same human immortalized keratinocyte HaCaT as control health skin model.

All cell lines were cultured in complete Dulbecco’s modified Eagle’s medium (DMEM; Hyclone, South Logan, UT) with high glucose (4.5 g/L), supplemented with 10% fetal bovine serum (FBS, HyClone), 2 mM–glutamine and 100 IU/mL penicillin/streptomycin (Invitrogen, Carlsbad, CA). Cells were kept at 37 °C in a humidified atmosphere with 5% CO_2_, until medium removal and harvesting by trypsin treatment.

The cells were passaged every 3–4 days at a sub–cultivation ratio of 1:5 and used within 5–20 passages. The cell pellet resulting from subsequent centrifugation for 5 min at 4000 rpm was then processed for genomic DNA extraction. The cells were lysed in hypotonic lysis buffer by repeated pipetting, incubated 15 min in ice, and centrifuged for 10 min at 2000 rpm and 4 °C, discarding the supernatant. The extraction of the genomic DNA has been performed by incubation for 1 h at 37 °C in 750 μL of nuclear lysis buffer, followed by a treatment with 250 μL of NaCl 6M and final centrifugation for 15 min at 2000 rpm and 4 °C. The supernatant containing genomic DNA was recovered and then precipitated adding EtOH 100%. The DNA pellet obtained by further centrifugation for 10 min at 2000 rpm and 4 °C was washed in EtOH 70%, centrifuged again for 10 min at 7500 rpm and 4 °C and re–suspended in DNase free H_2_O. Wash buffers generally contain alcohols and can be used to remove proteins, salts and other contaminants from the sample. The DNA concentrations, that was kept constant throughout the entire study at ca. 20 ng/μ L, were measured with a spectrophotometer (Eppendorf BioSpectrometer^®^ basic) by reading absorbance at 260 nm and 260/280 ratio, absorbance was checked to assess the purity of the DNA. The DNA purity was tested by spectrophotometer analysis, measuring the ratio of absorbances at 260/280 and at 260/230. In fact, the spectrophotometry is recommended for quantification analysis. This technique gives information on the nucleic acid concentration of the sample along with two quality ratios, 260/280 and 260/230. The 260/280 ratio reflects the purity of the DNA and RNA. At 260 nm nucleic acids are measured and at 280 proteins. The recommended 260/280 ratio should be between 1.8 and 2.1. If the ratio is lower, it may indicate the presence of protein or other contaminants that absorb at 280 nm. The 260/230 ratio is also used as a measure of nucleic acid purity. At 260 nm, nucleic acids are measured, and at 230 chemicals remaining in the sample from the isolation step are measured. The 260/230 values should be in the range of 2.0–2.2. If the ratio is lower than expected it may indicate the presence of contaminants, which absorb at 230 nm. The 260/280 and 260/230 ratios resulted 1.8 and 2, respectively, for each sample analyzed. For further details, the reader is referred to^12,14,15^.

#### Raman analysis

A DXR2xi Thermo Fisher Scientific Raman Imaging Microscope has been used to collect a Raman map of all the DNA drops deposited onto the nanostructured substrates and left to dry in air at Room Temperature. The maps have been collected by using a 532 nm laser source, with a 1 mW excitation power and a 50 × objective in a backscattering configuration.

Each spectrum composing the map resulted from 4 accumulations lasting 5 ms, and the map step size has been fixed in 4 μ so as to obtain 4000 spectra for each drop. The map dimension and step size have been established taking into account several specific requirements, such as the need to collect the same large number of spectra for each sample coming from the central part of the drop, in order to better exploit the SERS effect associated with the nanostructured substrate, and the fact that the spectra composing the map must come from points distant enough to be considered independent acquisitions for the classification methods. For each sample, HaCaT, SK-MEL-28, A375, CaCo-2, and HT29, the entire measurement process has been repeated 5 to 10 times.

### Data set and pre-processing

We performed a pairwise comparison of the experimental data by comparing each time two sets of Raman spectra coming from two different samples. More precisely, we considered six cases: (1) HaCaT vs. A375, (2) HaCaT vs. SK-MEl-28, (3) SK-MEl-28 vs. A375, (4) HaCaT vs. CaCo-2, (5) HaCaT vs. HT29, and (6) CaCo-2 vs. HT29. For each case the two involved samples are conventionally called *first* and *second* sample.

In each comparison the data set consisted of $N=4000$ spectra, 2000 for each sample. The spectra have been randomly chosen in the central part of the droplets to exploit maximally the interaction between the DNA molecules and the nanostructured substrate. Since the spectra are collected at points of the droplet at distance larger than, or equal to, 4 μm we assume that the data are independent^23^. To remove the influence of fluorescence on data analysis, a standardized background correction has been provided as an automatic output of the Raman apparatus and was identically performed on all the maps by polynomial fitting (order 3). Large part of the collected spectra share similar features. The few which are substantially different from the others have been considered outliers and removed from the analysis. To do this, we built a decision surface by adding and subtracting three times the (point–wise) empirical standard deviations to the average spectra and discarding those spectra featuring at least one point outside the decision surface. Moreover, we smoothed the data by filtering the original raw spectra with the Savitzky–Golay algorithm^25^ (see also^26^) over a window of 90 data points treated as convolution coefficients.

### Statistical approaches

We propose five different models to classify the spectra. Two are rather simple, essentially geometric, and based on the evaluation of global characteristics of the spectra: the average and the $\ell ^2$ distance from the average. The other three are more sophisticated, they take into consideration the local properties of the spectra, and are based on the PCA analysis, the average pooling of the spectra, and the propagation of the spectra through a 1-D Convolutional Neural Network (CNN).

The spectra are split into training and test sets, the first used to tune the parameters of the models here proposed, the second to validate them. We deal with a binary target variable *W*, whose outcomes 1 and 0 correspond to the DNA molecules of the first and the second sample, respectively.

#### Logistic regression on global average (LRA)

The first model we proposed is based on a very simple idea, i.e., the global mean of the spectral intensity is exploited as the unique predictor of a logistic regression model. Thus, the mean value provides a representation of the spectra in both the LW and HW regions. This method does not involve highly detailed data analysis and is meant to provide a basic tool to classify Raman Spectra. In particular, it is of interest to check whereas such a simple approach can be sufficient to identify properly tumoral DNA. The model is penalized by means of an $L^2$ regularization with setting 1 as shrinkage parameter. The model is validated by means of a ten-fold cross-validation, and its goodness is assessed by the average score of the ROC–AUC (Receiver Operating Characteristic–Area Under the Curve) curve over the ten cross-validation folds.

#### Evaluation of $\ell ^2$ distance (L2D)

The second method here proposed is based on the analysis of some geometric features of the whole considered portion of the spectra and has already been presented in^23^ and it can be described as follows. The training set is used to compute average spectra of the two samples, represented by the column vectors of $\mathbb {R}^p$, $h^1$ for the first sample and $h^2$ for the second one. Then, the $\ell ^2$ distance between both these averages and each spectrum belonging to the test set and represented by the *i*-th row of the data matrix $\textbf{X}$ is computed as follows,

1 $$\begin{aligned} d ^k(i) = \sum _{s=1}^p|x_{is}-h^k_s|^2 \;\;\; for\, k=1,2 .\end{aligned}$$

A classifier for each spectrum is then built thanks to the following outcome binary function

2 $$\begin{aligned} g_out (i) = \mathbb {I}\{\tau d ^1(i)\le (1-\tau )d ^2(i)\} , \end{aligned}$$

where $\tau \in [0,1]$ is an optimization parameter, again optimized as above with a ten-fold validation. The outcome is set as equal to 1 if the spectrum is identified as coming from the first sample.

#### Logistic regression on average pooling (LRP)

The third method here proposed is also based on a logistic regression model, where the input features are computed by applying the average pooling operator on the input spectra. Thus, the spectral domain of each spectrum is divided into non-overlapped and non-equispaced sub-domains and for each subset the mean value is computed. Such an approach aims to pre-process and represent the profile of each spectrum with a lower number of explanatory variables. Depending on the binary task to solve, a finer or coarser partitioning of the spectral domain can be chosen; here a four and three-feature representation has been set for the LW and HW regions, respectively.

Again, the logistic regression model is penalized by the $L^2$ regularization with the shrinkage parameter equal to 1 and the model validation follows a ten-fold cross-validation procedure.

The *permutation importance* technique^27^ is here used to investigate which input features mainly support the predictions of the logistic regression model. This technique is often employed to inspect if the random shuffling of one column feature can drastically decrease the accuracy of the model. Indeed, a random permutation in a column feature causes the break of the correlations between that specific explanatory variable and the target variables and produces a drop in the predictive performance of the model. To quantify the degradation of the accuracy due to the permutations on one column feature, we evaluate the difference between the ROC–AUC of the model and the average ROC–AUC after applying a finite number of permutations. Such a quantity is labeled as the *importance score* of one column feature. In this case, 30 distinct permutations on each column feature have been applied.

#### Logistic regression on PCA components (PCA)

We use the notation borrowed from^28^, Chapter 1 and Paragraph 8.2.1 and already used in^23^. Any single spectrum, in the considered interval, is represented as a column vector $x\in \mathbb {R}^p$. By collecting the *N* row vectors $x^\dag$, where $\dag$ denotes transpositions, we construct the $N\times p$ matrix $\textbf{X}$ which represents the entire data set in the considered interval. The *j*th column of $\textbf{X}$ is the collection of the *N* observations of the *j*th variable, namely, the intensity corresponding to the *j*th value of the Raman shift.

Thus, we compute the $N\times p$ matrix $\textbf{Y}$ by centering $\textbf{X}$ with respect to the columns (i.e., the Raman shift). A *principal components analysis* is then obtained by the eigendecomposition of the empirical covariance matrix ${\textbf {Y}}^\dag {\textbf {Y}}$^29^. The *principal components directions* $v_1,\dots ,v_p\in \mathbb {R}^p$ are computed and we call *i*-th *principal component loadings* the *p* elements of the column vector $v_i$. The projection $\textbf{z}_i=\textbf{Y}v_i\in \mathbb {R}^N$ is called *i*-th *principal component* (PC) of the data $\textbf{Y}$ and its variance of each PC is given by the corresponding eigenvalue. The variance concentrates on the first *m* principal components, allowing us to neglect in the next step all the other $p-m$ components. This is way we call “local” this method.

The selected first *m* principal components $z_1,\ldots ,z_m$ are thus interpolated to build a logistic regression model to estimate the probability mass function of the binary target variable *W* by

3 $$\begin{aligned} Pr (W=1|z_1,\ldots ,z_m)=\frac{e^{\beta _0+\sum _{i=1}^m\beta _iz_i}}{1+e^{\beta _0+\sum _{i=1}^m\beta _iz_i}} \;\; and \;\; Pr (W=0|z_1,\ldots ,z_m)=\frac{1}{1+e^{\beta _0+\sum _{i=1}^m\beta _iz_i}}, \end{aligned}$$

where $\beta _i\in \mathbb {R}$ for $i=0,\ldots ,m$. In this case, we consider an optimization parameter $\lambda \in [0,1]$ such that we associate the outcome for the binary variable $W=1$ to each set of components $z_1,\ldots ,z_m$ if $Pr (W=1|z_1,\ldots ,z_m)\ge \lambda$ and $W=0$ otherwise. The tuning parameter $\lambda$ is estimated by means of a ten-fold cross-validation procedure (see,^30^, Ch. 7).

The original sample is randomly partitioned into ten equally sized subsamples. A subsample is kept as test set, while the other nine ones are used as training data. Then the accuracy of both the methods are evaluated, while the cross-validation process is repeated ten times, paying attention to use each round a different subsample as test group. The ten results are then averaged to compute a single estimation. The advantage of this validation strategy is that all observations are used at the same time for both training and testing and each observation is used for testing exactly once.

#### 1-D convolutional neural network (CNN)

1-D CNN represents a type of feed–forward neural network designed to solve a broad class of classification tasks when the input features are precisely 1-D grid–structured data^31–34^. Such a class of models combines convolutional and max-pooling operators to encode the sequentiality of the patterns contained in the input data. As a result, the optimization of the weights defining the convolutional filters of the convolutional layers aims to give the most linearized latent representation of the input Time–Series. In our case, therefore, we regarded the spectra as some Time-Series whose “temporal evolution” takes place along the spectral domain.

Before being propagated thought the layers of the CNN model, the spectra are neither rescaled nor transformed furtherly. The model, therefore, is validated by means of ten-fold cross-validation. To assess the goodness of the model, we compute the ROC–AUC score on each fold; the average AUC score over all the cross-validation folds is to be intended as the goodness of the model. The Standard Error Mean is used to estimate the error on the average ROC–AUC score.

The design of our CNN is purely convolutional, i.e. it consists of a sequential combination of *Convolutional Layers* followed by *Max–Pooling Layers*. The non-linear activation function is embedded in the convolutional layer; in specific, we opted for a *softplus* function, i.e. $\phi (x) = \log {(1+\exp {(x)})}$. Dropout layers^35^ with dropout rate of 0.25 are also employed to contrast overfitting. Each convolutional layer possesses 64 filters whose convolutional masks have an amplitude of 3 pixels; the pooling size of the pooling layers is equal to 2. The sequence of convolutional and pooling layers is then repeated three times; the resulting feature map is therefore flattened by means of a *Flatten Layer*. Finally, the flattened feature map is propagated through one *Fully-Connected Layer* with 16 output nodes and softplus activation function. This particular layer returns the latent representation of the spectra. The latent representation is therefore propagated through a *Fully-Connected Layer* with a sigmoid activation function and one output node, that is the output node of the CNN. During the training phase, the ADAM^36^ algorithm is used to optimize the Binary-Cross Entropy loss function. The batch size and learning rate are set equal to 64 and 0.001, respectively.

As we know, feed-forward neural networks are usually regarded as black-box models whose activity cannot be expressed by means of closed forms. However, we can visualize the impact that a single input feature has on the final predictions by means of the Vanilla Gradient method^37^. Such a method allows the construction of *saliency maps* based on the evaluation of the gradients $\frac{\partial o}{\partial X_i}$, with *o* the output value of the CNN and $X_i$ the *i*-th input feature. The evaluation of the score-derivative $\frac{\partial o}{\partial X_i}$ is nothing but the change needed at the pixel $X_i$ to affect the class score the most^37^. Note that, in our case, $X_i$ is exactly the intensity of the *i*-th Raman shift. The visualization and the interpretation of saliency maps, however, can often suffer from scale problems. Such an inconvenience is often adjusted by applying a monotone function to map the values of the score derivative into the desired interval. We, therefore, opted for the empirical cumulative functions of the score-derivatives themselves, i.e. we construct a saliency map that is specific to the test set. Hence, we feed the CNN model with the instances of the test set; next, we backpropagate the output scores via the Vanilla Gradient method and finally apply the desired empirical distribution function on the score derivatives.

#### Differentiating tumor subtypes: unsupervised learning analysis

Before concluding this section, we present briefly some results related to the so-called unsupervised learning approach, used here to verify whether different tumor subtypes can be associated with varying features of *SERS*. In particular, we utilized the Spectral Clustering algorithm^38^ with the pairwise cosine kernel as an affinity matrix. Therefore, we designed two unsupervised analyses only to compare two classes of tumor subtypes, i.e., A375-SKMEL28 and CaCo-HT29.

The data partitioning performed by the Spectral Clustering algorithm exploits the property of the spectrum of the connection graph associated with the data. In practice, one builds a graph between all the instances, with the edges representing the affinity between the instances themselves. In our case, the affinity (or adjacency) matrix of the graph, which we shall denote with $\kappa$, is given by

4 $$\begin{aligned} \kappa (X, Y) = \frac{<X, Y>}{\sqrt{<X, X> <Y, Y>}}; \end{aligned}$$

with *X*, *Y* two generic array-like instances, and $<\cdot , \cdot>$ denoting the scalar product. It is important to mention that before passing the instances through the algorithm, we standardized each spectrum, i.e., we removed the mean value and make the standard deviation unitary.

To assess the goodness of the Spectral Clustering, we compared the labels predicted with the corresponding true labels. Therefore, we utilized Matthews’s correlation coefficient (MCC)^39^ and visualized the confusion matrix. We recall Matthews’s correlation coefficient, denoted here as ${\text {MCC}}$, can be calculated directly from the confusion matrix, namely,

5 $$\begin{aligned} {\text {MCC}} = \frac{n_{00} n_{11} - n_{01} n_{10}}{\sqrt{(n_{00}+n_{10})(n_{00}+n_{01})(n_{11}+n_{10})(n_{11}+n_{01})}}; \end{aligned}$$

with the generic $n_{ij}$ denoting the number of instances with with true label *i* and predicted label *j*.

## Results and discussion

**Figure 1:**
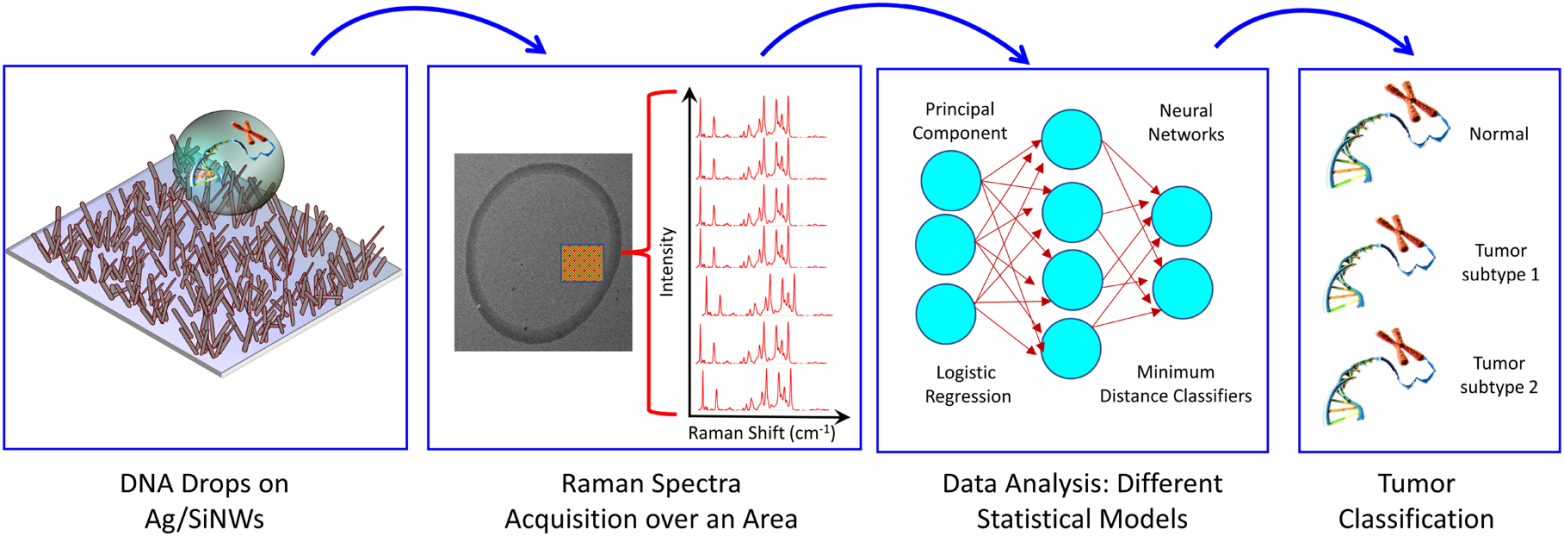
Graphical representation of the phases of the diagnostic procedure.

**Figure 2:**
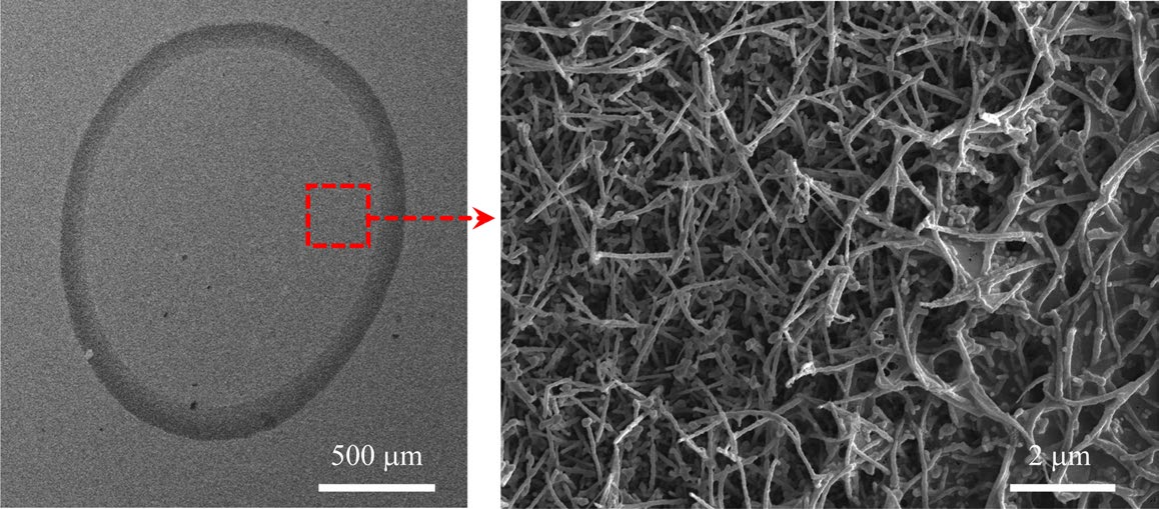
SEM images of a representative DNA drop on Ag/SiNW after water evaporation (left panel) and high magnification image of the area inside the red square (right panel).

**Figure 3:**
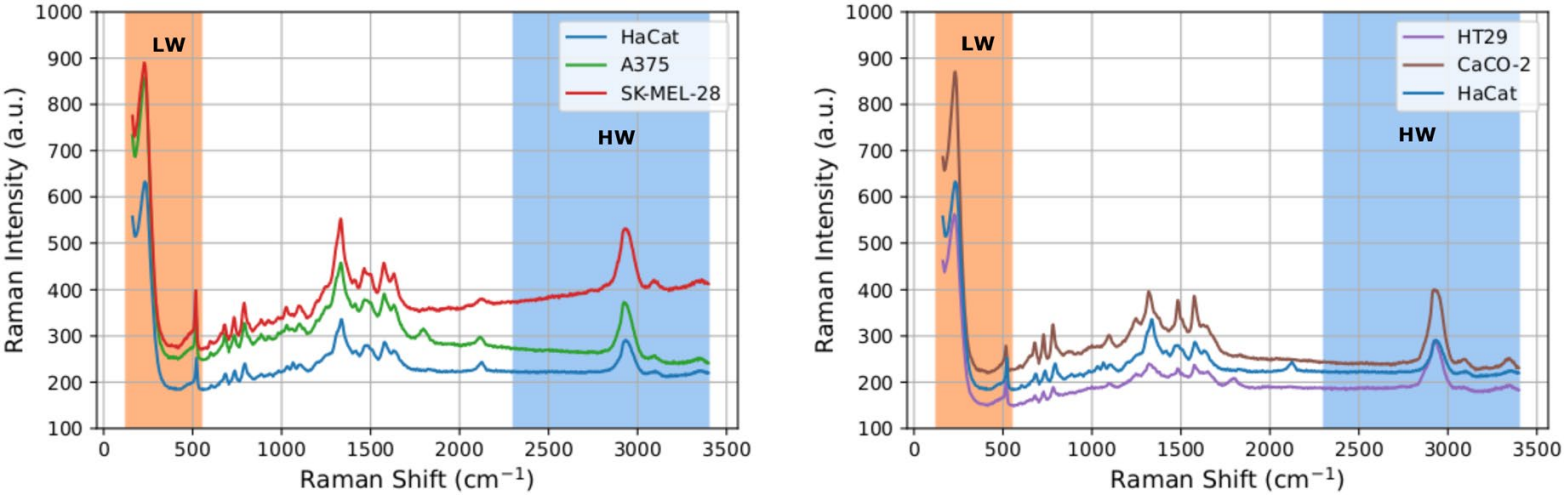
Left: average Raman spectrum for the HaCaT (blue), A375 (green), SK-MEL-28 (red) samples. Right: average Raman spectrum for the HaCaT (blue), CaCo-2 (brown), HT29 (purple) samples.

As depicted in Fig. 1, which schematically represents the subsequent phases of the diagnostic procedure, drop casting has been used to deposit healthy and cancer DNA solutions on the Ag/SiNWs platform. Figure 2 (left panel) shows a representative dried DNA drop characterized by the typical coffee–ring pattern. The magnified SEM image of the area in the red square, reported in the right panel, shows the morphology of the disordered mat of Ag/SiNWs, which are 2–3 μm long and have diameters ranging from 80 to 150 nm. Furthermore, it is possible to observe a slight sticking of the wires due to the presence of adsorbed DNA. Raman maps were collected in the central part of the drops, to exploit maximally the interaction between the DNA molecules and the nanostructured substrate. Actually, as already observed in Refs.^11,15^, the region inside the annular ring enables a more sensitive SERS detection with respect to the coffee ring, where the analyte agglomeration is maximum. The small thickness of the analyte layer just inside the ring allows more of the deposit to be in direct contact with the Ag/SiNWs permitting the enhancement of the Raman signal, while on the annular ring the Ag/SiNWs are essentially buried under a thick layer of analyte so that the SERS effect is limited. Figure 3, left and right panels, report the average Raman spectra calculated over the entire maps for HaCaT with the two melanoma phenotypes, and HaCaT with the two colon cancer phenotypes, respectively. Some specific features can be recognized: (i) the bands directly ascribed to the DNA molecules, i.e., those located between 600 and 1200 cm^−1^ due to aromatic in-plane bending vibrations of the bases and stretching vibrations of the phosphate moiety; (ii) the peak at about 234 cm^−1^, associated with the metal-nitrogen (Ag–N) stretching vibration mode of the generated surface bond between the deposited nucleotides and the Ag coverage of the NWs^40–43^; (iii) the peak at about 514 cm^−1^, originated from the SiNWs themselves, that produce a detectable Si signal even through the Ag coating; (iv) the pronounced large band at about 2934 cm^−1^, corresponding to the stretching vibrations of the CH_2_ and CH_3_ groups^4^.

The aforementioned Raman features are thus related not only to intrinsic chemical characteristics of the DNA molecules, but also to their physical properties, which influence the molecule arrangement on, and interaction with, the NWs, carrying important diagnostic information. In fact, the band around 234 cm^−1^ takes into account the specific DNA molecule adsorption on the nanostructured Ag surface through the N atoms of the basis rings, so that unrepaired oxidative DNA damage, or a different stiffness, can influence the Raman signal at that band. The Si peak at 514 cm^−1^ coming from the substrate provides information on the surface distribution of the molecules: a different DNA conformation results in a diverse substrate coverage and causes a consequent variation of the peak intensity. Finally, the band at 2934 cm^−1^, comprising contributions coming from C–H vibrations, is clearly conditioned by the degree of DNA methylation. On the basis of these considerations, we performed our statistical analysis by focussing the attention on two principal spectral ranges: the *low wavenumbers* (LW) region consisting of $p=221$ spectral points with wavenumber ranging from 125.25 cm^−1^ to 549.27 cm^−1^ (orange selection in Fig. 3) and the *high wavenumbers* (HW) region consisting of $p=570$ spectral points with wavenumbers from 2303.16 cm^−1^ to 3399.83 cm^−1^ (light blue selection in Fig. 3).

We first discuss our results for the melanoma data, to concentrate subsequently on the colon tumor ones. Here, our aim is to show that the applied classification methods achieve a very good accuracy when applied to different experimental settings and targets.

**Table 1:** AUC values for the five methods proposed in Section “Statistical approaches” for the three melanoma related cases listed at the beginning of Section “Data set and pre-processing” in the low wavenumber (LW) and high wavenumber (HW) spectral regions.

	LRA	L2D	LRP	PCA	CNN
HaCaT vs. A375 LW	0.99	0.82	0.99	0.99	0.99
HaCaT vs. A375 HW	0.97	0.90	0.96	0.99	0.96
HaCaT vs. SK-MEL-28 LW	0.99	0.89	0.99	1.00	0.99
HaCaT vs. SK-MEL-28 HW	0.99	0.99	0.99	0.99	0.99
SK-MEL-28 vs. A375 LW	0.61	0.62	0.88	0.93	0.90
SK-MEL-28 vs. A375 HW	0.95	0.94	0.99	0.99	0.99

The first column specifies the case and the spectral region. In the following five columns the AUC values are reported for the methods: logistic regression on global average (LRA), computation of $\ell ^2$ distance (L2D), logistic regression on average pooling (LRP), logistic regression on PCA components (PCA), 1-D convolutional neural network (CNN).

**Figure 4:**
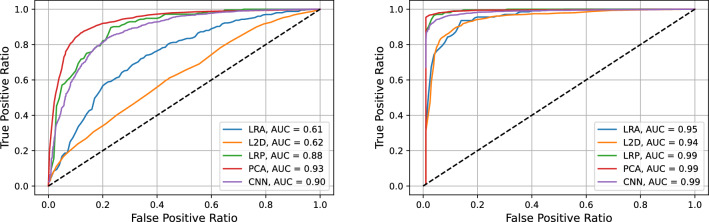
SK-MEL-28 vs. A375 comparison. ROC graphs for the methods logistic regression on global average (blue), computation of $\ell ^2$ distance (orange), logistic regression on average pooling (green), logistic regression on PCA components (red), 1-D convolutional neural network (purple) in the low (left) and high (right) wavenumber regions.

**Figure 5:**
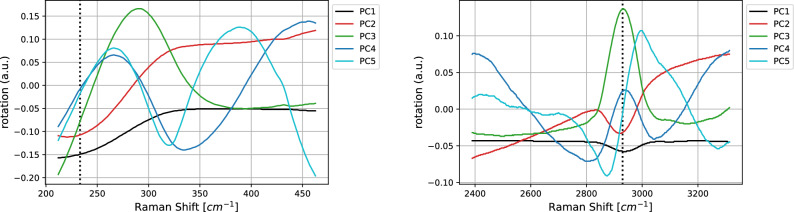
SK-MEL-28 vs. A375 comparison. Loadings of the first five PCA components in the LW (left) and HW (right) wavenumber regions. Black, red, green, blue, and light blue, respectively, for components from one to five.

**Table 2:** SK-MEL-28 vs. A375 comparison.

	PC1	PC2	PC3	PC4	PC5
LW region					
Standard deviation	7684.2752	251.63904	136.90699	51.71480	36.92418
Proportion of variance	0.9984	0.00107	0.00032	0.00005	0.00002
Cumulative proportion	0.9984	0.99948	0.99980	0.99984	0.99987
HW region					
Standard deviation	5128.2426	190.61525	53.14363	28.83052	19.76634
Proportion of variance	0.9984	0.00138	0.00011	0.00003	0.00001
Cumulative proportion	0.9984	0.99983	0.99994	0.99997	0.99998

Standard deviation, proportion and cumulative proportion of variance related to the first five PCA components in LW and HW regions, respectively.

### Analysis of melanoma data

In order to evaluate the ability of the methods proposed in Section “Statistical approaches” to classify the Raman spectra we first report in Table 1 the AUC values obtained with the different methods for the HW and LW regions of the spectra. The associated ROC graphs are reported in figure 4.

We first note that the low wavenumber part of the spectra allows to distinguish healthy (HaCaT) and tumor (SK-MEL-28 or A375) samples with all the proposed methods. The less performing is the one based on the computation of the $\ell ^2$ norm. On the other hand, this spectral region does not seem to ensure a good classification of tumor subtypes, indeed, in the comparison between the SK-MEL-28 and the A375 data only the PCA method is able to perform with an AUC larger than 0.9. The other two local methods work reasonably well with a 0.88 AUC, while the global methods performance is absolutely poor.

The tumor samples are, on the contrary, very well classified and distinguished with all the proposed methods applied to the high wavenumber part of the spectra.

The performance of the local methods is generally better than that of the global ones, but the reason why they are particularly useful is that they provide us with a detailed information about the physical and the chemical phenomena which allow the classification of the tumor subtypes.

Thus, we restrict our discussion to the SK-MEL-28 vs. A375 comparison and show how our statistical analysis provides information at the physical and chemical level.

**Figure 6:**
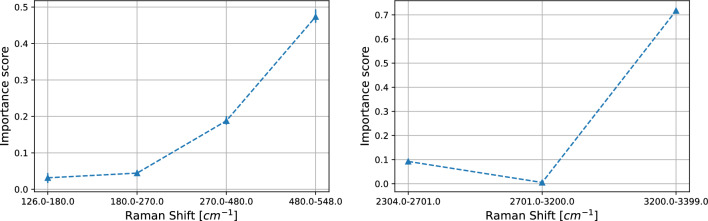
SK-MEL-28 vs. A375 comparison. Importance scores (see Section “Statistical approaches” ) for the logistic regression on average pooling for LW (left) and HW (right) regions. On the x–axis the sub-regions and on the y-axis the average importance scores. The error bars represent the 95% confidence interval.

**Figure 7:**
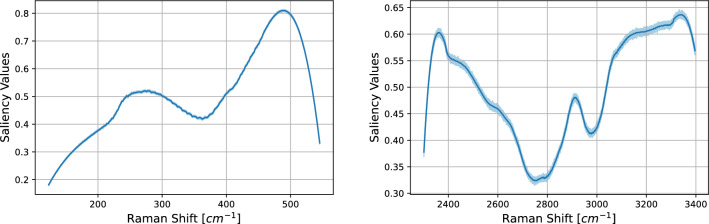
SK-MEL-28 vs. A375 comparison. Saliency maps via Vanilla Gradient algorithm (see Section “Statistical approaches” ) of the 1D-CNN models for both LW (left) and HW (right) regions. On the x-axis the spectral domain while on the y-axis the average saliency values. The light blue and the light red areas represent the 95% confidence interval.

**Figure 8:**
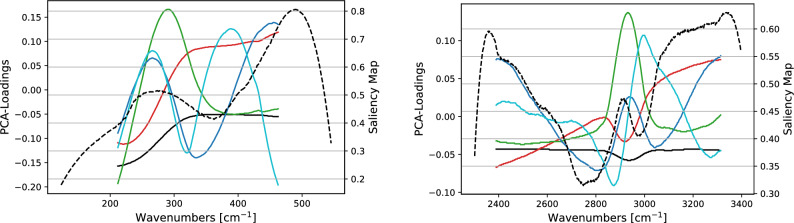
SK-MEl-28 vs. A375 comparison. We report the PCA loadings using the same color code as in Fig. 5 and the salience map (dashed line) for the 1-D CNN applied to the spectra obtained by subtracting their mean value.

In Fig. 5 we report the loadings computed for the first five PCA components, while Table 2 collects their values of standard deviation, proportion, and cumulative proportion of variance in both the HW and LW regions. Note that in both the regions the cumulative variance associated with these components exceeds the value 99.9%, with most of the variance related to the first principal component PC1. As shown in Fig. 5, the contributions of the input variables to the first principal component—the black line—is almost constant in both the regions, with the exception for the peak at 234 cm^−1^ (LW region) and 2930 cm^−1^ (HW region). A peculiar behavior in correspondence of both the peaks is shown also by the second component, enforcing the hypothesis that these two peaks are the most relevant to achieve binary classification within this model.

In Fig. 6 the average importance scores of the logistic regression on average pooling are shown. As one can see, the LW region is particularly sensitive at 480–548 cm^−1^ with importance scores of 0.48. That region contains the peak associated to the silicon nanowires (514 cm^−1^, see Fig. 3), whose classification role is likely associated to the different arrangement of DNA molecules on the nanostructures, resulting in a diverse surface coverage and SERS enhancement factor, that in turn causes a different weight of the signal coming from the nanowires with respect to the DNA one. Instead, other main physical characteristics, still representing the interaction between the samples and the substrate, as the broad peak around 234 cm^−1^ (see Fig. 3) do not support the high performance of the logistic regression. In the HW region one can see that the binary problem is solved by exploiting information laying in sub-band at 3200–3399 cm^−1^. Note that the more relevant regions for average pooling are not related to the peaks crucial for the classification based on the PCA. In our opinion this is due to the peculiarities of the two different model. Average pooling exploits equisized intervals in the frequency domain; within each interval each frequency contributes equally to the construction of the logistic regression. On the other hand, PCA builds subsets of frequencies based their importance to justify the dispersion of the data (i.e., the contribution to the variance). Then, in the first approach, the importance of the peaks, which are crucial for the PCA, could be mitigated by the surrounding frequencies.

In Fig. 7 the saliency maps of the 1D–CNN models are shown. The saliency map presents two broad peaks around 285 cm^−1^ and 490 cm^−1^ with saliency values of 0.52 and 0.82 respectively. Both these regions are not centered on some crucial wavenumbers such as 234 cm^−1^ or 512 cm^−1^; where physico–chemical spectral lines are usually located. When dealing with the HW region, one can see that the saliency map reveals a flat region with saliency 0.6 at the right of the peak of the CH vibrations, i.e., 3030–3300 cm^−1^.

In Fig. 8 we report the loadings related to the PCA components and the salience map for the 1-D CNN (left and right vertical axes respectively) run on the spectra after having subtracted their mean. As far as LW is concerned, on the one hand 1-D CNN does not identify the region surrounding 200 cm^−1^ as predictive, while the one after 300 cm^−1^ is considered salient. On the other hand, the first PCA component detects that the spectrum intensity decreases until 300 cm^−1^ to become then almost constant. Other PCAs, such as the third and the fourth components, provide a better representation of the characteristics at 230 cm^−1^. It has to be remarked that the proportion of variance explained by these two components is 2 orders of magnitude smaller than the one associated to the first PCA component.

Regarding HW, the 1-D CNN model is capable to put in evidence the peak associated to the CH vibrations even with a salience 0.6, not particularly high. Within the PCA framework, also in this case the first components describes mainly a scaling factor, even if—at least marginally—also the band of the CH vibrations is represented. Higher components, for example the fourth and the fifth, put more in evidence the band located at about 2930 cm^−1^.

**Table 3:** As in Table 1 for the three colon tumor cases listed at the beginning of Section “Data set and pre-processing”.

	LRA	L2D	LRP	PCA	CNN
HaCaT vs. CaCo-2 LW	0.98	0.90	0.99	0.96	0.99
HaCaT vs. CaCo-2 HW	0.97	0.73	0.99	0.98	0.99
HaCaT vs. HT29 LW	0.75	0.90	0.93	0.99	0.93
HaCaT vs. HT29 HW	0.97	0.98	0.99	1.00	0.94
CaCo-2 vs. HT29 LW	0.94	0.87	0.99	1.00	0.97
CaCo-2 vs. HT29 HW	0.83	0.93	0.93	0.99	0.99

**Figure 9:**
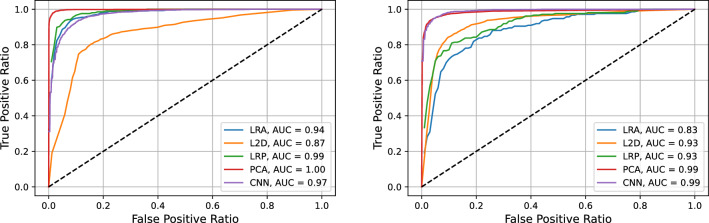
As Fig. 4 for the case CaCo-2 vs. HT29.

**Figure 10:**
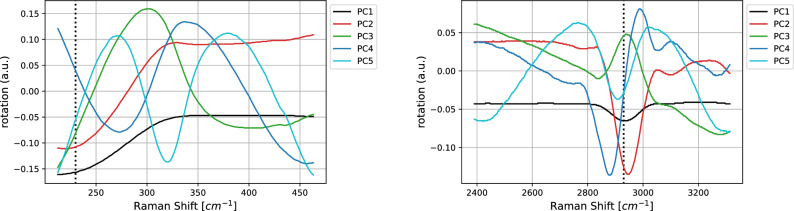
As Fig. 5 for the case CaCo-2 vs. HT29. Black, red, green, blue, and light blue identify principal components from one to 5 respectively.

### Analysis of colon tumor data

As explained above, we tested the robustness of the proposed methods by applying our analysis to a data set obtained by using different tumor samples, namely, the colon tumor cells listed as cases 4–6 in Section “Data set and pre-processing”. Although the average spectra compare each other differently with respect to the melanoma case, see Fig. 3, we will show that our techniques perform well also in this case.

We report in Table 3 the AUC values obtained with the different methods for the high and low wavenumber regions of the spectra. The associated ROC graphs are reported in Fig. 9.

We note that, although the global methods do not perform perfectly in some of the cases, the local ones achieve absolutely remarkable performances in classifying tumor vs. healthy samples and also in classifying the two different tumor phenotypes (CaCo-2 vs. HT29 comparison).

In the remaining part of this section we restrict our discussion to the CaCo-2 vs. HT29 comparison and show how our statistical analysis provides information at the physical and chemical level.

**Figure 11:**
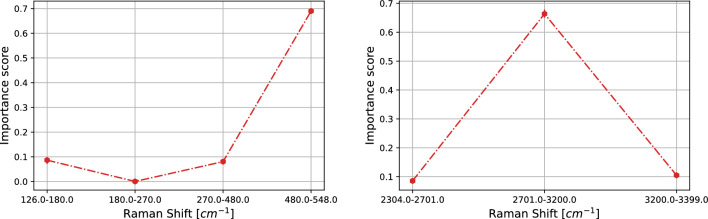
As Fig. 6 for the case CaCo-2 vs. HT29.

**Figure 12:**
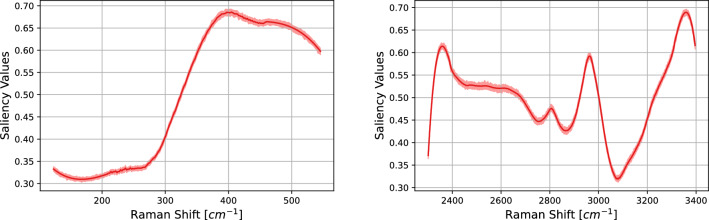
As Fig. 7 for the case CaCo-2 vs. HT29.

In Fig. 10 we report the loadings computed for the first five PCA components. Regarding the LW region (left panel), the contributions of the input variables to the first principal component—the black line—are larger at lower frequencies, to became then constant at higher ones. In the same region, the second principal component is characterized by similar contributions, but opposite in sign. Finally, in the third component a peak is showed at about 310 cm^−1^. In the HW region (right panel), the contributions are almost constant for all the involved frequencies, with the exception of a small peak at 2930 cm^−1^. Other higher peaks characterize in the same region also the second and third principal components, suggesting its importance to achieve binary classification.

In Fig. 11 the average importance scores of the logistic regression on average pooling are shown. As for the melanoma case, the LW region is particularly sensitive at 480–548 cm^−1^ with larger importance scores of order 0.69. Similarly to the SK-MEl-28 vs. A375 case, the broad peak around 234 cm^−1^ (see Fig. 3) does not support the high performance of the logistic regression. In the HW region, instead, the binary problem is solved by exploiting information laying in different sub-bands with respect to the ones relevant in the melanoma case. More specifically, a high importance score of 0.65 is found in the sub-band 2701–3200 cm^−1^, i.e., where is located the broad peak representing the vibrational modes of CH-groups (usually represented by a broad peak centered at 2930 cm^−1^; see, Fig. 3).

In Fig, 12 the saliency maps of the 1D–CNN models are shown. As one can see, the cases SK-MEL-28 vs. A375 and CaCo-2 vs. HT29 support in a different way the highly accurate predictions of the CNN model. As already noted above, the saliency map of case SK-MEL-28 vs. A375 presents two broad peaks around 310 cm^−1^ and 490 cm^−1^. Likewise, the saliency map of case CaCo-2 vs. HT29 reveal a salient region at 350–514 cm^−1^. When dealing with the HW region, one can see that the saliency map of case CaCo-2 vs. HT29 can reveal a narrow salient region in correspondence of 2930 cm^−1^ (a peak with saliency value 0.6).

**Table 4:** MCC for different tumor subtypes and wavenumber regions.

Case	MCC
A375-SKMEL28, HW	0.9
A375-SKMEL28, LW	0.56
CaCO-HT29, HW	0.66
CaCO-HT29, LW	0.83

**Figure 13:**
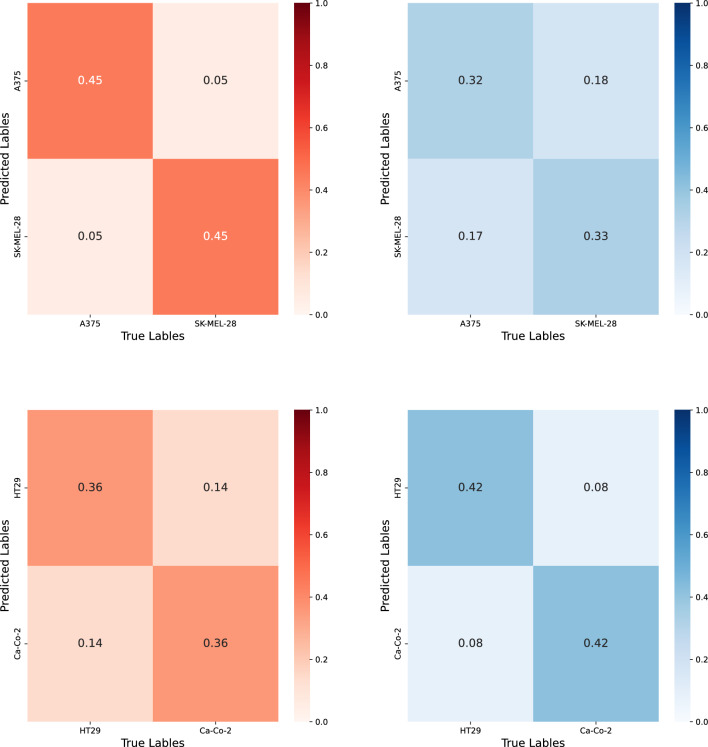
Confusion matrixes with rows representing predicted labels and columns the true labels. Each cell expresses the density of instances associated with each couple of predicted-true labels. (**a**) case A375-SK-MEL-28 in HW region, (**b**) A375-SKMEL28 in LW region, (**c**) CaCo-2-HT29 in HW region, (**d**) CaCo-2-HT29 in LW region.

As far as the differentiation of tumor subtypes by means of Unsupervised Learning Analysis is concerned, as shown in Table 4, high correlation values between the predicted and true labels can be found for the case A375-SK-MEL-28 in the HW region (MCC = 0.9) and the case CaCo-2-HT29 in the LW region (MCC = 0.83). When considering the confusion matrix collected in Fig. 13a–d), the A375-SK-MEL-28 in the HW region, 90% of instances could be partitioned according to the actual labeling, (see Fig. 13a), while in the LW region this percentage corresponds to 65% (see Fig. 13b). As far as CaCo-2-HT29 is concerned, while in the HW region we obtain 71% of correctly labeled instances (see Fig. 13c), in the LW region, this percentage is equal to 83% (see Fig. 13d).

## Conclusions

In this paper we have demonstrated via a thorough statistical analysis that Raman mapping obtained by dropping genomic DNA on disordered Ag/SiNWs can be used to classify different subtypes of tumors both for melanoma and colon cancer.

We have developed several statistical approaches to classify the experimental data showing that both the global and local methods are able to distinguish the healthy and malignant DNA molecules through a different interaction of these molecules with the Ag/SiNWs platform, mainly affecting the low wavenumber region of the analysed spectral range. In addition, the local methods achieve absolutely remarkable performances in classifying the diverse tumor phenotypes. Indeed, 1-D CNN model takes advantage of its pattern recognition activity to capture the characteristic bands associated with the CH vibrations, located in the high wavenumber region of the analysed spectral region, by allowing to separate different phenotypes for both melanoma and colon cancer.

A similar result is achieved by means of the method based on a PCA decomposition, which exploits subsets of relevant frequencies to perform a logistic regression. In our opinion, the better performance of local methods can be explained since they select preliminarily relevant subsets of frequencies, optimizing the classification procedure and discarding all the redundant information. Indeed, local methods take advantage of the fine structure of the peaks sequence, whereas the global ones rely on the mere computation of an average quantity.

We highlight that the discrimination of DNA coming from distinct malignant phenotypes occurs without any knowledge of the basis sequencing, unlike most of the present biochemical methods. Rather, the classification ability of the platform appears to be mainly due to samples’ differences involving the CH vibrations, so that it can be inferred the methylation degree plays an important role in the diagnostic process thanks to its effects on the conformational properties of DNA. Ultimately, our results suggest that the analysis of Raman spectra of genomic DNA directly dropped onto disordered Ag/SiNWs with 1-D CNN and PCA algorithms provides a rapid, simple, and accurate discrimination of the cancer subtypes by offering a powerful and effective guidance in the patient personalized treatment.

## Data Availability

The datasets used and analysed during the current study are not public. They are available from Valentina Mussi (valentina.mussi@cnr.it) on reasonable request.
